# SNP-Based Genome-Wide Association Mapping of Pollen Viability Under Heat Stress in Tropical *Zea mays* L. Inbred Lines

**DOI:** 10.3389/fgene.2022.819849

**Published:** 2022-03-16

**Authors:** Zubair Ahmed, Maria Khalid, Abdul Ghafoor, Muhammad Kausar Nawaz Shah, Ghazala Kaukab Raja, Rashid Mehmood Rana, Tahir Mahmood, Addie M. Thompson

**Affiliations:** ^1^ Department of Plant Breeding and Genetics, Pir Mehar Ali Shah Arid Agriculture University, Rawalpindi, Pakistan; ^2^ Crop Disease Research Institute, National Agricultural Research Center (Pakistan), Islamabad, Pakistan; ^3^ Institute of Biochemistry and Biotechnology, Pir Mehr Ali Shah Arid Agriculture University, Rawalpindi, Pakistan; ^4^ Pakistan Agricultural Research Council, Islamabad, Pakistan; ^5^ Department of Plant, Soil and Microbial Sciences, Michigan State University, East Lansing, MI, United States

**Keywords:** maize, extreme temperature, genetic mapping, pollen sterility, yield, flowering time

## Abstract

Global environmental changes with more extreme episodes of heat waves are major threats to agricultural productivity. Heat stress in spring affects the reproductive stage of maize, resulting in tassel blast, pollen abortion, poor pollination, reduced seed set, barren ears and ultimately yield loss. As an aneamophelous crop, maize has a propensity for pollen abortion under heat stress conditions. To overcome the existing challenges of heat stress and pollen abortion, this study utilized a broad genetic base of maize germplasm to identify superior alleles to be utilized in breeding programs. A panel of 375 inbred lines was morpho-physiologically screened under normal and heat stress conditions in two locations across two consecutive planting seasons, 2017 and 2018. The exposure of pollen to high temperature showed drastic decline in pollen germination percentage. The average pollen germination percentage (PGP) at 35 and 45°C was 40.3% and 9.7%, respectively, an average decline of 30.6%. A subset of 275 inbred lines were sequenced using tunable genotyping by sequencing, resulting in 170,098 single nucleotide polymorphisms (SNPs) after filtration. Genome wide association of PGP in a subset of 122 inbred lines resulted in ten SNPs associated with PGP35°C (*p* ≤ 10^−5^), nine with PGP45°C (*p* ≤ 10^−6^–10^−8^) and ten SNPs associated with PGP ratio (*p* ≤ 10^−5^). No SNPs were found to be in common across PGP traits. The number of favorable alleles possessed by each inbred line for PGP35°C, PGP45°C, and the PGP ratio ranged between 4 and 10, 3–13 and 5–13, respectively. In contrast, the number of negative alleles for these traits ranged between 2 and 8, 3–13 and 3–13, respectively. Genetic mapping of yield (adjusted weight per plant, AWP^−1^) and flowering time (anthesis-silking interval, ASI) in 275 lines revealed five common SNPs: three shared for AWP^−1^ between normal and heat stress conditions, one for ASI between conditions, and one SNP, CM007648.1-86615409, was associated with both ASI and AWP^−1^. Variety selection can be performed based on these favorable alleles for various traits. These marker trait associations identified in the diversity panel can be utilized in breeding programs to improve heat stress tolerance in maize.

## Introduction

Global maize production exceeds 1,108 million tons, making it one of the most widely grown cereal crops around the world (FAOSTAT, 2020). Maize production is continuing to increase by 7.4% annually in Pakistan, but changing climate conditions and global warming pose serious threats to maize productivity around the world, particularly in the Indian subcontinent. Since 1850, each of the last 4 decades have been successively warmer, and global surface temperature has risen 1°C during 2011–2021 relative to 1850–1900 (IPCC, 2021). Each degree-Celsius increase in global temperature will reduce the global maize yield by 7.4% (Zhao et al., 2017). It is predicted that extreme heat stress during anthesis will reduce maize production by 45% from 1980 to 2080 (Deryng et al., 2014).

Maize plants are particularly sensitive to heat stress at the flowering stage, causing more yield reduction than during the grain filling stage. (Zhang et al., 2013). The reproductive tissues are the most sensitive parts of the plant, so a few degrees increase in temperature at the flowering stage can cause devastating losses in grain yield (Lobell et al., 2015) High temperatures (33–40°C) also have a negative effect on light capture, harvest index, and grain and biomass yields. Breeding for climate resilient maize is the only solution to overcome climatic adversities as predicted by climate change models. Evaluating maize germplasm for pollen viability is an effective approach for the development of climate resilient hybrids. Phenotypic studies along with genomic information can reduce breeding time by pinpointing suitable genomic regions and improving selection efficiency (Kulwal et al., 2014).

The genome wide association study (GWAS) has emerged as a powerful approach for identifying genes underlying complex morphological traits (Wang et al., 2016; Yano et al., 2016; Mwadzingeni et al., 2017; Nadeem et al., 2021), in which a diverse natural population is used to detect the statistical association between markers and traits. Next generation sequencing techniques like genotyping-by sequencing (GBS) scan the genome and generate millions of SNPs, providing dense genome coverage for the identification of desirable marker-trait associations in different plant species (Atwell et al., 2010; Huang et al., 2010; Tian et al., 2011). In maize, genome wide association studies have been employed for flowering time (Salvi et al., 2007), kernel size and weight (Li et al., 2010), kernel quality (Manicacci et al., 2009), drought tolerance (Thirunavukkarasu et al., 2014), and various other target traits (Xue et al., 2013), including root system architecture under drought stress (Zaidi et al., 2016; Darlene et al., 2018), and plant leaf angle and lodging and heat stress tolerance in subtropical maize (Longmei et al., 2021). Though mapping has been conducted for flowering time and yield, no GWAS is previously reported in maize considering pollen viability directly as the target trait.

The objectives of this study were: 1) evaluation of a maize diversity panel consisting of exotic and indigenous maize inbred lines for heat stress tolerance, considering pollen viability, yield, and ASI as the target traits, and 2) identification of marker-trait associations and quantification of their effect on the traits.

## Materials and Methods

### Plant Material and Experimental Design

A maize diversity panel composed of 375 diverse inbred lines was used for this study. The diversity panel includes 103 exotic lines, 283 indigenous lines, and three check cultivars: Haq Nawaz Gold, FH-1898, and CML161. The diversity panel was formulated by collection of diverse maize genotypes from exotic sources (CIMMYT, Pakistan) and from plant genetic resources (PGRI), NARC, Pakistan. The details of the diversity panel are given in Supplementary Table S1. The trial was planted for two spring seasons in 2017 and 2018 each with normal (1st week of March) and late (1st week of April) sowing dates at both the National Agricultural Research Center (NARC), Islamabad, Pakistan, and Peshawar, Pakistan.

Two different sowing dates were used so that the flowering periods would be synchronized to both a normal temperature and a heat stress temperature in each year. Significant temperature differences at flowering time were observed in normal and stressed sowing trials. The normal sowing trials flowered during the end of May, and temperatures at the flowering stage (VT) were in the range of 29–35°C. The stressed sowing trials flowered during early to mid-July, and the temperatures at VT stage were in the range of 38–45°C.

Seeds were hand sown in single row plots of length 4 m using a dibbler at a depth of 3 cm. Row to row distance was maintained at 75 cm while plant to plant distance was maintained at 25 cm such that each plot contained 16 plants, and fertilizer was applied at the rate of 200:100:100 NPK. Manual thinning and weeding operations were performed to retain optimum plant numbers and weed control in the experiments. The same experimental plots were used for both years in both locations.

### Phenotyping

Phenotypic data of various agronomic traits were collected according to published maize descriptors (CIMMYT/IBPGR 1991). To assess the relationship of other phenotypic traits to pollen viability, days to anthesis (DTA), days to silking (DTS), anthesis silking interval (ASI), and adjusted weight per plant (AWP^−1^) were collected from 5 plants for each of the 375 varieties. At tasseling (VT) stage, DTA, DTS and ASI were recorded, while yield was measured at the R6 stage. Assessment of pollen germination was carried out on a subset of 122 lines using pollen germination media (PGM) proposed by Dresselhaus et al. (2011), using pollen collected from the first (non-stressed) planting of the NARC trail in 2017. Fresh pollen was collected between 7am with 70% relative humidity and 8am with 63% relative humidity. Anthers, insects and other contaminants were cleaned by sieving the collected pollen. Pollen from each inbred line was dusted on six disposable petri plates each containing 20 ml of PGM. Out of six, three petri plates were placed at 35°C and three at 45°C in a growth chamber under dark conditions with 70% relative humidity. The pollen was observed under a light microscope and photographed. The pollen germinated percentage (PGP) was recorded by counting viable pollen grains out of 100 pollen grains from each plate at two different temperature regimes i.e., 35 and 45°C. From these values, three traits were recorded: PGP 35°C, PGP 45°C, and the ratio between the two.

### Statistical Analysis

Descriptive statistics of various morphological traits were calculated by using Microsoft Excel 365 data analysis tools. The recorded data for all the parameters were used for the analysis of variance (ANOVA) (Finney, 1964). Mean square values of each source of variation were used to calculate the genotypic variance σ^2^G and phenotypic variance σ^2^P, and Broad-sense heritability was calculated using formula H^2^ = σ^2^G/σ^2^P. Field experiment data was analyzed as a randomized design in R software (R Core Team, 2018).

### Genotypic Data

Genotypic data of 275 maize accessions were generated by Freedom Markers from fresh tissue via tunable genotyping by sequencing (Ott et al., 2017) with the restriction enzyme Bsp1286I. Briefly, genotypes were sequenced using an Illumina HiSeq X instrument, and reads were aligned to the *Zea mays* AGPv4 reference genome after de-barcoding and trimming of reads. SNP calling was conducted using only those reads that aligned to a single location in the reference genome. Initially, nearly 2.7 million SNPs were identified, with a mean missing data rate per SNP site of 64%. From these, a high-quality set of 496,740 SNPs in which each marker was genotyped in at least 50% of the samples was generated and referred to hereafter as MCR50 SNPs. Each of these SNPs were supported on average by 20 tGBS reads per SNP per genotyped sample. Finally, imputation was performed on the MCR50 SNPs using BeagleV4.1 (Browning and Browning, 2007; Browning and Browning, 2016) with 50 phasing iterations and other default parameters. This marker set was further filtered to exclude sites with a minor allele frequency <5% in the set of phenotyped varieties, yielding a final marker dataset of 170,098 sites across 262 varieties. These were relatively evenly distributed across the 10 chromosomes (Figure 2).

Population structure and kinship matrices of the sets of 122 (for pollen traits) and 275 (for other traits) maize accessions were generated within the rMVP program (Yin et al., 2021) in R software (R Core Team, 2018).

### Association Mapping

Phenotypic data of all accessions were checked for outliers, and all high-quality data was used for association studies. The FarmCPU algorithm (Liu et al., 2016) within the rMVP package (Yin et al., 2021) of R (R Core Team, 2018) was used for association mapping, with the kinship matrix (k matrix) and an optimal number of principal components to account for population structure (Q matrix) and prevent false-positive associations. Quantile-quantile (Q-Q) plots of estimated versus observed *p*-values for marker-trait associations were produced to assess model fit and select the appropriate number of principle components for each trait. Bonferroni corrections at 0.05 yielded a significance threshold of 2.94 × 10^−7^ (0.05 threshold divided by 170,098 markers). Because Bonferroni can be overly stringent due to linkage, all SNPs with *p*-values lower than 10^−5^ are reported here as potential associations as a hypothesis-generating approach.

## Results

### Morphological Analysis and Correlation Among Traits

Morphological data from the maize diversity panel was combined across the 2 years in the study. Phenotypic analysis showed considerable variation for all traits under both normal and heat stress conditions (Table 1). The diversity panel was evaluated based on five agro-morphological traits to inform the assessments of pollen viability, the major trait under study. Average pollen germination percentage (PGP) at 35°C temperature was 40.3% with a range of 0%–93.7%. In contrast, the average PGP at 45°C temperature was 9.7% with a range of 0%–57%. The average decline in PGP from 35 to 45°C was 30.6%. The mean AWP^−1^, DTA, DTS, and ASI was 37.5 g, 62.5, 64.6, and 2.9 days, respectively, which were significantly reduced to 35.5 g, 47.4, 50, and 2.4 days under heat stress (Table 1). ANOVA results also indicated that significant variation was observed among the genotypes under both normal and heat stress conditions in both years.

**TABLE 1:** Descriptive statistics of various geomorphological traits in subset of 122 diverse maize inbred lines planted under normal and heat stress conditions across 2 years (spring 2017 and 2018) at Islamabad (NARC).

Trait	Condition	Mean	SD	Min	Max	σ^2^ P	σ^2^ G	H^2^
PGP35°C	**Normal**	40.30	29.60	0.00	93.70	1749.08***	851.79***	0.49
AWP^−1^		37.50	9.10	8.00	85.00	1735.46***	1512.98***	0.87
DTA		62.50	6.90	51.00	80.70	161***	94.7***	0.59
DTS		64.60	7.70	38.30	84.00	194.60***	177.5***	0.91
ASI		2.90	0.70	1.70	6.70	0.95**	0.31**	0.32
PGP45°C	**Stress**	9.70	11.70	0.00	57.00	282.17**	112.67**	0.40
AWP^−1^		35.50	20.00	0.00	122.30	123.71***	54.23***	0.44
DTA		47.40	0.71	30.00	7.60	42***	15***	0.36
DTS		50	0.70	34.00	72.60	50.5***	10.5***	0.21
ASI		2.90	0.04	1.33	7.20	0.85	0.30	0.35

* = 0.05% significant, *** = 0.001% significant.

PGP, Pollen germination percentage at 35 and 45°C, AWP^−1^: adjusted weight per plant, DTA, days to anthesis; DTS, days to silking; ASI, anthesis silking interval; SD, standard deviation; Min, Minimum; Max, Maximum; σ^2^ P, phenotypic variance; σ^2^ G, genotype variance; H^2^, heritability.

Broad-sense heritability of all traits was estimated from the NARC location and ranged between 0.32 for ASI to 0.91 for DTS under normal sowing conditions which was significantly reduced to 0.21 for DTS to 0.44 for AWP^−1^ under heat stress (Table 1).

### Relationships Among Traits

Correlation analysis was performed on the combined data of normal and stress trials. The PGP at 35°C showed positive correlation with AWP^−1^ (r = 0.54), DTA (r = 0.49) and DTS (r = 0.14), but PGP at 45°C showed negative correlation with AWP^−1^ (r = −0.3), DTA (r = −0.07) and DTS (r = −0.1). On the other hand, DTA and DTS showed negative correlation with yield under heat stress conditions, at (r = −0.64) and (r = −0.76), respectively (Table 2).

**TABLE 2:** Correlation of traits under normal (above diagonal) and heat stress (below diagonal) conditions; diagonal shows the correlation between normal and heat stressed conditions for each trait.

Traits	AWP^−1^	DTA	DTS	ASI	PGP (35°C)
AWP^−1^	0.87	−0.64	−0.76	0.62	0.54
DTA	0.96	0.58	−0.1	−0.03	0.49
DTS	0.52	0.7	0.59	0.06	0.14
ASI	0.11	0.9	0.93	0.23	−0.09
PGP (45°C)	−0.3	−0.07	-0.1	0.14	0.55

AWP^−1^, adjusted weight per plant; DTA, days to anthesis; DTS, days to silking; ASI, anthesis silking interval; PGP, Pollen germination percentage at 35 and 45°C.

### Genome Wide Association Study

**FIGURE 1:**
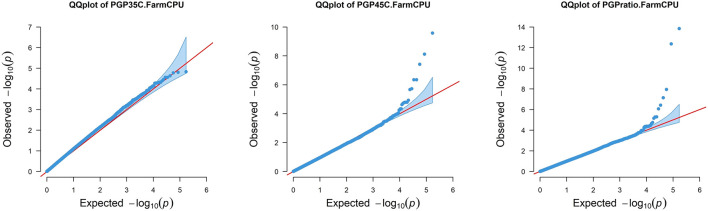
Quantile-quantile (Q-Q) plots using Q + K FarmCPU model, showing comparative rise of expected −log_10_ *p*-values on x-axis against observed −log_10_ *p*-values on y-axis for each trait. PGP35°C: Pollen Germination percentage at 35°C; PGP45°C: Pollen Germination percentage at 45°C and PGP Ratio: Pollen Germination percentage ratio between 35 and 45°C.

**FIGURE 2:**
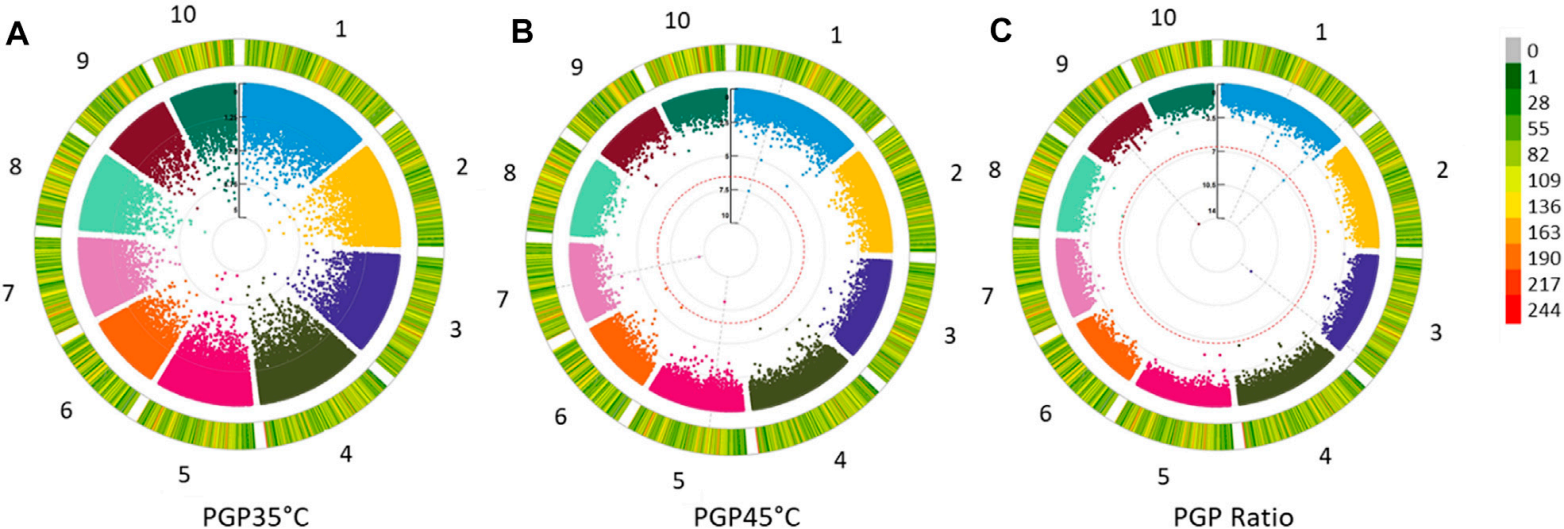
Circular Manhattan plots revealing significant QTLs **(A)** PGP35°C: Pollen Germination percentage at 35°C; **(B)** PGP45°C: Pollen Germination percentage at 45°C and **(C)** PGP Ratio: Pollen Germination percentage ratio between 35 and 45°C in 122 maize inbred lines. The −log_10_ *p*-values are used to plot against each of the 10 chromosomes represented in 10 different colors. The outer most circle indicates the marker density and side legend bar indicates the number of SNPs within a 1 MB window size.

Pollen Germination Percentage PGP 35°C, PGP 45°C and the ratio between the two were used for GWAS. In addition, GWAS was also performed for AWP^−1^ and ASI for two locations across two seasons and two sowing dates. Q-Q plots representing the distribution of SNP significance for each PGP trait are represented in Figure 1. A total of 29 potential SNPs were identified that may have association with the target traits (Figure 2; Table 3). Despite the smaller subset of varieties used to map PGP, GWAS identified 14 highly significant SNPs associated with PGP traits: one with PGP35°C on chromosome 2, nine with PGP45°C on chromosomes 1,4,5 & 9, and four with PGP ratio on chromosomes 2 and 9.

**TABLE 3:** Genome wide association studies (GWAS) results indicating SNPs associations with the target traits under various conditions using 122 (PGP) or 262 (AWP^−1^ and ASI) diverse maize inbred lines.

Traits	Total SNPs	1	2	3	4	5	6	7	8	9	10	*p* Value
PGP35°C	10		3	2	3	1					1	10^−5^–10^−6^
PGP45°C	9	4			1	3				1		10^−6^–10^−8^
PGP Ratio	9	1	2	3						3		10^−5^–10^−7^
AWP^1^.NARC.2017.D1	1	1										10^−5^–10^−6^
AWP^1^.NARC.2017.D2	9		4	1	2				1	1		10^−6^–10^−10^
AWP^1^.NARC.2018.D1	14		4	1	3	1			4		1	10^−6^–10^−7^
AWP^1^.NARC.2018.D2	13	5		1	1	2	2			2		10^−6^–10^−10^
AWP^1^.Peshawer.2017.D1	4	1	1								2	10^−5^–10^−6^
AWP^1^.Peshawer.2017.D2	4	3							1			10^−5^–10^−6^
AWP^1^.Peshawer.2018.D1	5	1		1			2		1			10^−5^–10^−6^
AWP^1^.Peshawer.2018.D2	9	2		1	1		2		1	1	1	10^−5^–10^−7^
ASI.NARC.2017.D1	2					1			1			10^−5^–10^−6^
ASI.NARC.2017.D2	11	4			1	1	1	1	2		1	10^−6^–10^−8^
ASI.NARC.2018.D1	9		1	1		4			2		1	10^−6^–10^−7^
ASI.NARC.2018.D2	3		2				1					10^−6^–10^−8^
ASI.Peshawer.2017.D1	8			3	1	2				1	1	10^−6^–10^−8^
ASI.Peshawer.2017.D2	13	5	2		3				1	1	1	10^−6^–10^−7^
ASI.Peshawer.2018.D1	11	3	1	1	5			1				10^−6^–10^−7^
ASI.Peshawer.2018.D2	10	1	1	3	1	1	1	1	1			10^−6^–10^−11^
Total	154	31	21	18	22	16	9	3	15	10	9	

No common association of SNPs were found for the PGP traits under study. SNP CM007650.1-89641171 located on 89.64 MB position of chromosome 1 showed the highest (0.25) R^2^ values, associated with PGP45°C. Among each trait various SNPs with highest R^2^ values were observed. Trait PGP35°C was associated with SNP CM007648.1-75060024 located on chromosome 2 which also had an R^2^ value of 0.25. PGP45°C also showed association with SNP CM007650.1-89641182located on chromosome 1 with the highest R^2^ value of 0.25. In the case of PGP ratio, SNP CM007649.1-225376365 located on chromosome 3 showed the highest R^2^ value of 0.24. PGP45°C showed the lowest *p*-value of (*p* ≤ 10^−8^) with SNP CM007650.1-89641171 located on chromosome 1 shown in Table 4 and Supplementary Table S2.

**TABLE 4:** Common SNPs significantly associated within and between target traits in diverse maize inbred lines.

Trait	SNP	Chromosome	Position	Position (MB)	MAF	R^2^	*p*-value
AWP^1^.Peshawer.2017.D1	CM007647.1-19525844	1	19525844	19.525844	0.485	0.16	5.32E-06
AWP^1^.Peshawer.2017.D2	CM007647.1-19525844	1	19525844	19.525844	0.23	0.23	4.46E-07
AWP^1^.Peshawer.2018.D1	CM007650.1-26720973	3	26720973	26.720973	0.47	0.25	9.60E-06
AWP^1^.Peshawer.2018.D2	CM007650.1-26720973	3	26720973	26.720973	0.49	0.24	7.38E-08
AWP^1^.Peshawer.2018.D1	CM000786.4-148468397	10	148468397	148.468397	0.37	0.13	1.40E-07
AWP^1^.Peshawer.2018.D2	CM000786.4-148468428	10	148468428	148.468428	0.43	0.23	6.65E-06
ASI.Peshawer.2018.D1	CM007647.1-258148529	1	258148529	258.148529	0.41	0.32	3.99E-06
ASI.Peshawer.2018.D2	CM007647.1-258192870	1	258192870	258.19287	0.455	0.12	1.08498E-06
ASI.Peshawer.2018.D2	CM007648.1-86615409	2	86615409	86.615409	0.47	0.24	2.13E-08
AWP^1^.NARC.2017.D2	CM007648.1-86660602	2	86660602	86.660602	0.47	0.25	1.27E-07

Cumulative Effect of Favorable and Unfavorable Alleles on PGP35°C, PGP45°C and PGP, ratio.

A total of 59 SNPs were associated with AWP^−1^ (Supplementary Figures S1, S2; Supplementary Table S1). Of these, only one SNP, CM007647.1-160363841 located on chromosome 1, was associated with AWP^−1^ at NARC in 2017 on planting date 1 (normal sowing). Fourteen SNPs located on chromosomes 2 (4), 3 (1), 4 (3), 5 (1), 8 (4), and 10 (1) showed significant association with AWP^−1^ at NARC in 2018 on planting date 1. Similarly, 4 SNPs located on chromosomes 1 (1), 2 (1), and 10 (2), and 5 SNPs located on chromosomes 1 (1), 3 (1), 6 (2), and 8 (1) were found to be associated with AWP^−1^ at Peshawar in 2017 date 1 and 2018 date 1, respectively. Four SNPs located on chromosomes 1 (3) and 8 (1), and 9 SNPs located on chromosomes 1 (2), 3 (1), 4 (1), 6 (2), 8 (1), 9 (1), and 10 (1) showed association with AWP^−1^ at Peshawar in 2017 date 2 and 2018 date 2, respectively. Out of 59 SNPs, 18 were highly significant (*p* ≤ 10^−6^) (Table 3; Supplementary Table S2).

For anthesis silking interval (ASI), 67 SNPs were associated (Supplementary Figures S3, S4; Supplementary Table S2). Two SNPs located on chromosomes 5 (1) and 8 (1), and 9 SNPs located on chromosomes 2 (1), 3 (1), 5 (4), 8 (2), and 10 (1) showed significant association with ASI at NARC in 2017 for planting date 1, and NARC in 2018 data 1, respectively. At Peshawar in 2017 and 2018 date 1, 8 SNPs on chromosomes 3 (3), 4 (1), 5 (2), 9 (1), and 10 (1), and 11 SNPs on chromosomes 1 (3), 2 (1), 3 (1), 4 (1), 5 (1), 6 (1), 7 (1), and 8 (1), respectively, showed association with ASI. Under heat stress conditions (date 2) in Peshawar in 2017 and 2018, 13 SNPs located on chromosomes 1 (5), 2 (2), 4 (3), 8 (1), 9 (1), and 10 (1), and 10 SNPs on chromosomes 1 (1), 2 (1), 3 (3), 4 (1), 5 (1), 6 (1), 7 (1), and 1 (1), respectively, showed associations with ASI. Out of a total of 67 SNPs, 26 were found to be highly significant (*p* ≤ 10^−6^) (Table 3; Supplementary Table S2).

### Cumulative Effect of Favorable Alleles on Traits

**FIGURE 3:**
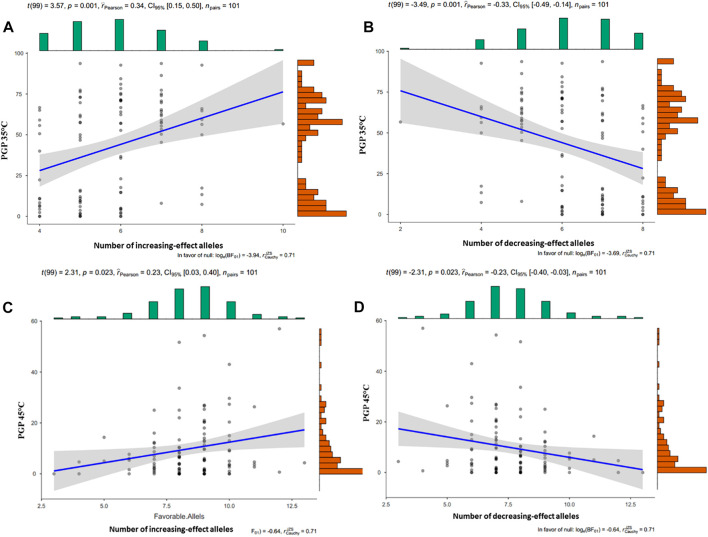
Cummulative effect of the number of favorable **(A)** and unfavorable **(B)** alleles on PGP 35°C, and of the favorable **(C)** and unfavorable **(D)** alleles on PGP45°C. The dots represent the mean values. The scatter plot shows PGP on the y-axis and number of alles on the x-axis.

The number of favorable alleles for PGP traits in each inbred line was investigated. For PGP35°C, this number ranged between 6 and 95 favorable alleles for each inbred line (Figures 3A,B). Similarly, the number of favorable alleles per line for PGP45°C ranged between 2 and 98 (Figures 3C,D).

## Discussion

Agro-morphological screening showed that the diversity panel behaved differently under normal and heat stress conditions. The main trait under study was pollen viability, and as expected, it was adversely affected by heat stress. The pollen viability was estimated in terms of pollen germination percentage (PGP). At 35°C the mean value of PGP was 40.30% with minimum PGP 0% and maximum PGP 93.70%, which was significantly reduced by 9.70% mean value and 0 and 57% minimum and maximum PGP respectively at 45°C. This is due to the fact that pollen germination of maize is hindered above 38°C Sánchez et al. (2014). The heat stress reduces the starch molecules in number and size within the pollen grain (Wang et al., 2019). Microspores exposed to heat stress during microsporogenesis leads to microspore abortion and pollen sterility (Begcy and Dresselhaus, 2018). Phenological traits like DTA, DTA and ASI were adversely affected by heat stress as significant reduction of mean DTA and DTS was observed in the diversity panel under heat stress as compared to normal sowing trials. The phenological development process of various crops is affected by high temperatures (Zhang et al., 2013).

In contrast, ASI is increased under heat stress compared to normal conditions; this was well explained by Cicchino et al. (2010). AWP^−1^ was the key indicator of plant response to heat stress in terms of major economic return. The mean AWP^−1^ was 37.5 g under normal conditions which was reduced to 35.50 g under heat stress conditions. Another important observation was that many of the inbred lines in the diversity panel produced baren plants under heat stress conditions. This is due to the fact that even a small increase (1.5°C) in temperature has a very significant negative effect on crop yield (Warland et al., 2006). An increase in average seasonal temperature by 1°C decreases the grain yield of cereal by 10 percent (Wang et al., 2012). Under heat stress conditions assimilatory capacity is reduced due to reduction in photosynthesis and increased maintenance respiration costs, resulting in productivity losses in wheat and maize crops (Reynolds et al., 2007).

In the case of maize, higher temperature (33–40°C) has a negative effect on light capture, harvest index, grain and biomass yield. At flowering, heat stress causes more yield reduction as compared to the grain filling stage (Zhang et al., 2013).

Broad-sense heritability estimates were moderate for traits under normal sowing trials, but were significantly reduced under heat stress conditions. This might be due to the fact that inbred lines were influenced by the adverse environment and affected the expression of traits. Our results were strongly in agreement with Noor et al., 2019 and Longmei et al., 2021. Correlation analysis revealed high positive correlation of AWP^−1^ with DTA (r = 0.96) and DTS (r = 0.52), and negative correlation with PGP 45°C (r = -0.3). DTA (r = 0.9) and DTS (r = 0.93) have high correlation with ASI under heat stress. PGP 35°C showed positive correlation with AWP^−1^ (r = 0.54), DTA (r = 0.49), and DTS (r = 0.14), and negative correlation with ASI (r = −0.09) under normal sowing conditions. The correlation analysis suggests that the traits under study might be influenced by the same genomic regions. Our studies are in coherence with previous studies (Xue et al., 2013; Wang et al., 2016; Zaidi et al., 2016). Further, overall time to flowering (DTA, DTS) seemed to be more indicative of yield (AWP^−1^) than PGP under heat stress. It is interesting that the association of DTA and DTS with yield (AWP^−1^) reverses directions under normal and heat stress conditions, perhaps emphasizing the importance of developmental stage on the impact of extreme weather conditions.

Association studies of PGP traits was carried out, and we considered all SNPs below 10^−5^ as candidates for future studies. Though this portion of the experiment was conducted on a reduced subset of 122 diverse inbred lines, 29 putative SNPs were found to be associated with the target traits at this significance level. Several studies have reported the association of traits with SNPs in maize using GWAS analysis (Xue et al., 2013; Wang et al., 2016; Carlos et al., 2019; Longmei et al., 2021). Because pollen germination percentage is a complex and time-consuming trait to score, this study was the first to our knowledge to map this trait in diverse exotic and indigenous maize inbred lines. Unique loci were identified that did not correspond with other physiological traits, introducing the possibility to investigate different genetic loci for heat stress adaptation traits in maize improvement.

GWAS of AWP^−1^ at two locations, years, and sowing dates in 275 lines revealed 59 MTAs. Of these, 24 were identified under normal sowing conditions (date 1), and 35 under heat stress conditions (date 2). Most of the SNPs that were identified were unique to a specific location and year, once again emphasizing the role of the environment and highlighting the need for validation of significant loci prior to their application in a breeding program.

In this study, three SNPs were identified as associated with AWP^−1^ in more than one condition, validating our results. In each case, these SNPs were associated with AWP^−1^ at Peshawar in 2017 on both date 1 and 2:

- SNP CM007647.1-19525844 on chromosome 1 at 19.5 MB
- SNP CM007650.1-26720973 on chromosome 3 at 26.7 MB
- SNP CM000786.4-148468397 on chromosome 10 at 148.5 MB

Though these SNPs were specific to location and year, they significantly impacted yield in both the normal and heat stress planting dates, indicating an overall contribution to fitness in that environment as opposed to a mechanism of heat stress tolerance specifically.

Association studies of ASI at two locations, years, and sowing dates identified a total of 67 SNPs distributed across nine chromosomes. Under normal sowing conditions, 30 MTAs were identified, while 37 SNPs showed significant association with ASI under heat stress conditions. Distribution of these traits across locations, years, and chromosomes are in Supplementary Table S2. As with AWP^−1^, most SNPs identified were associated with only one environment. However, one SNP, CM007647.1-258148529 on chromosome 1 at 258 MB was found to be commonly associated with ASI at Peshawar in 2018 at both sowing dates, validating its association with ASI.

Finally, one SNP, CM007648.1-86615409 on chromosome 2 at 86.6 MB was found to be associated with two different traits: ASI at Peshawar in 2018, and AWP-1 at NARC in 2017, both under heat stress conditions. This locus may be of interest to pursue in future studies or for breeding heat tolerant varieties, as it was validated across traits, locations, and years as significantly impacting both yield and ASI in heat stress conditions specifically.

The results indicate that different regions on chromosomes are responsible for the expression of various traits under study. This might be due to a difference in gene regulatory mechanisms under heat stress conditions compared to normal conditions, or perhaps different subsets of a greater regulatory network become critical in different specific environments. It is also possible that a more powerful experiment (more lines, environments, replications) may have identified additional shared loci. Still, SNPs were identified as significantly associated with the traits of interest, and a subset were validated. Based on these associations, candidate genes can also be identified and investigated for their roles in stress signaling. In previous studies, 206 significant SNPs were found associated with 115 candidate genes for drought tolerance and related traits (Wang et al., 2016). Sixty-seven SNPs were significantly associated with root structural traits as reported by Zaidi et al. (2016). Similarly, another study reported by Frey et al. (2015) revealed 607 heat responsive genes. Longmei et al. (2021) reported 46 SNPs associated with target traits under heat stress conditions in maize, and none of the SNPs were colocalized with multiple traits under heat stress conditions.

To understand the effect of combined favorable and unfavorable alleles on PGP35°C, PGP45°C, and PGP Ratio, the number of increasing effect alleles were investigated. Alleles corresponding to higher average PGP were considered favorable alleles, and those corresponding to lower average PGP were considered unfavorable alleles. The number of PGP 35°C increasing effect alleles ranged from 4 to 10, and the number of PGP 35°C decreasing effect alleles ranged between 2 and 8, Figures 3A,B. In the case of PGP 45°C, increasing effect alleles ranged from 3 to 13 and decreasing effect alleles ranged from 6 to 13, Figures 3C,D. The PGP ratio increasing and decreasing effect alleles ranged from 5 to 12 and 6–13, respectively.

## Conclusion

Identification of maize genomic regions responsible for better crop performance under heat stress conditions is essential for the selection of inbred lines and development of heat tolerant hybrids. In this study, pollen viablility is considered as a major factor, reponsible for yield losses under heat stress conditions. Importantly, the exotic genotypes were not very adaptive, and the local germplasm showed resiliance aginst heat stress conditions. There was significant reduction in PGP from 40.30% at 35°C to 9.70% at 45°C. This reduction in PGP showed negative correlation with yield contributing traits as well. GWAS indicated 14 significant SNPs associated with PGP35°C, PGP45°C and PGP ratio. Further work in identification of candidate genes using these significant SNPs is required to fully elucidate the role of these genomic regions in heat stress tolerance.

## Data Availability

The original contributions presented in the study are included in the article/Supplementary Material, further inquiries can be directed to the corresponding authors.
