# Supplementary material for: SNP-Based Genome-Wide Association Mapping of Pollen Viability Under Heat Stress in Tropical Zea mays L. Inbred Lines
Source: Front Genet. 2022 Mar 16;13:819849. doi: 10.3389/fgene.2022.819849 (PMC8966704; doi:10.3389/fgene.2022.819849)
Supplement: Supplementary file 2 [file Presentation1.PPTX]

## Slide 1
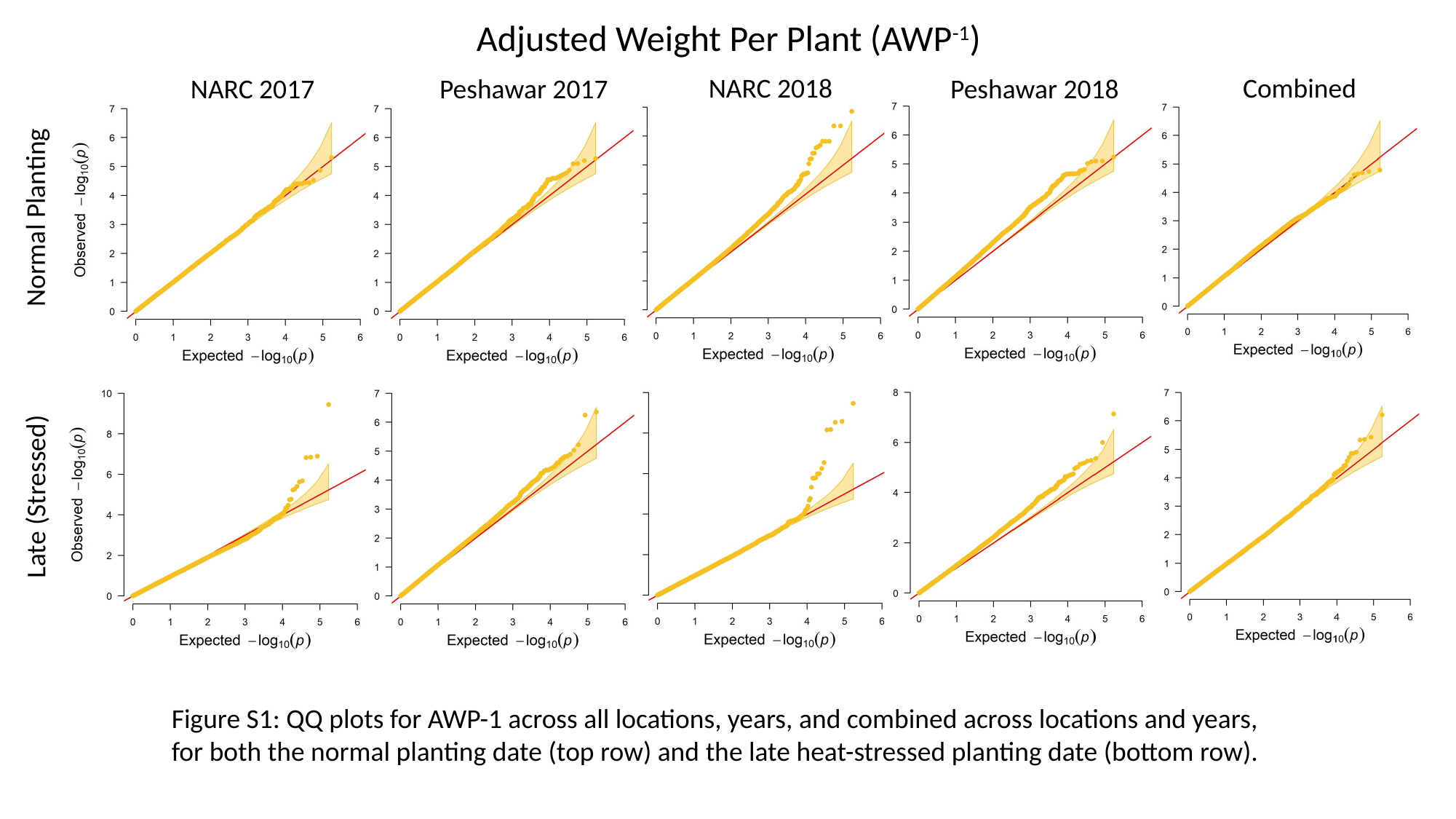

Adjusted Weight Per Plant (AWP-1)
NARC 2018
Combined
NARC 2017
Peshawar 2017
Peshawar 2018
Normal Planting
Late (Stressed)
Figure S1: QQ plots for AWP-1 across all locations, years, and combined across locations and years, for both the normal planting date (top row) and the late heat-stressed planting date (bottom row).

## Slide 2
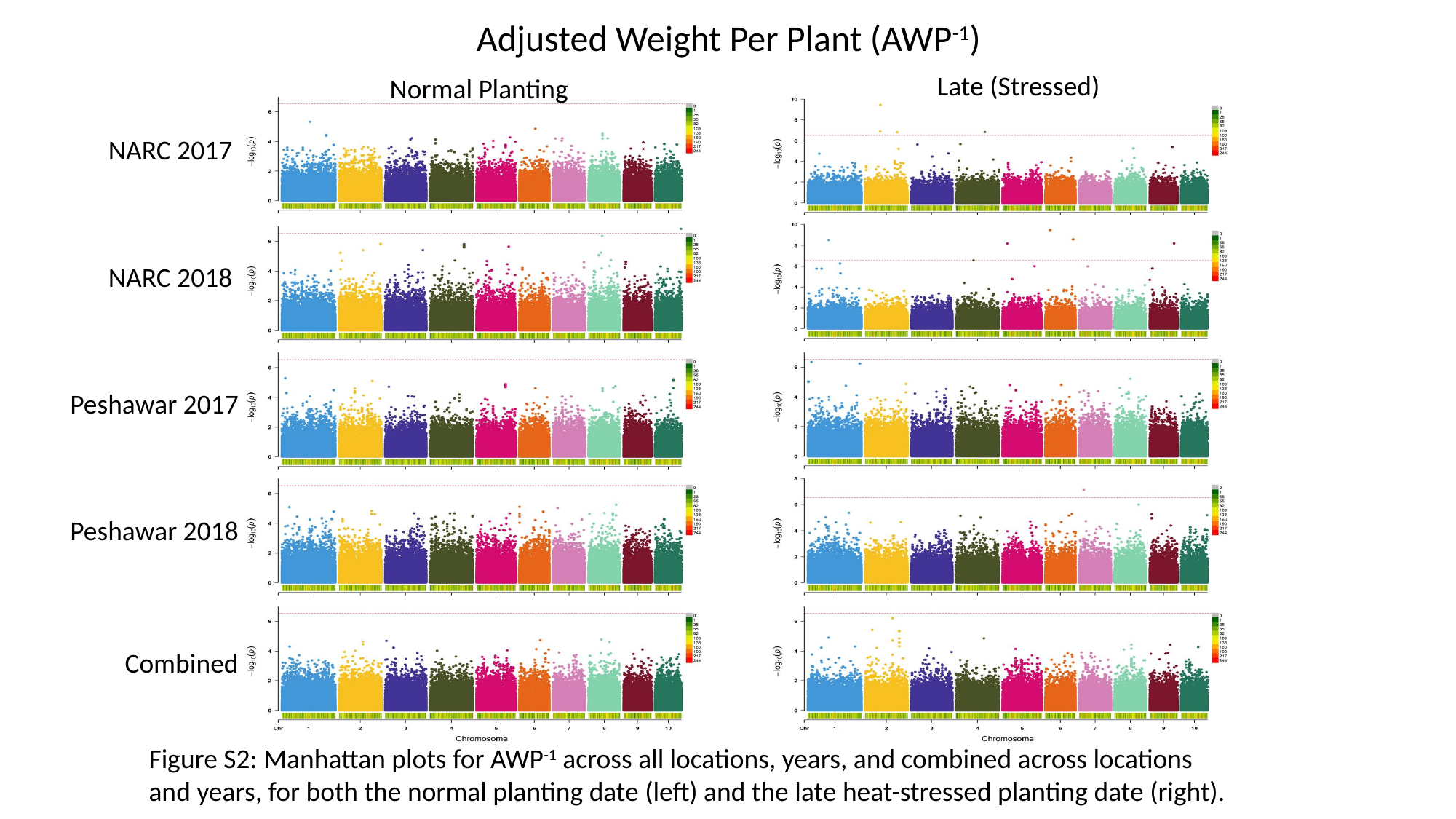

Adjusted Weight Per Plant (AWP-1)
Late (Stressed)
Normal Planting
NARC 2017
NARC 2018
Peshawar 2017
Peshawar 2018
Combined
Figure S2: Manhattan plots for AWP-1 across all locations, years, and combined across locations and years, for both the normal planting date (left) and the late heat-stressed planting date (right).

## Slide 3
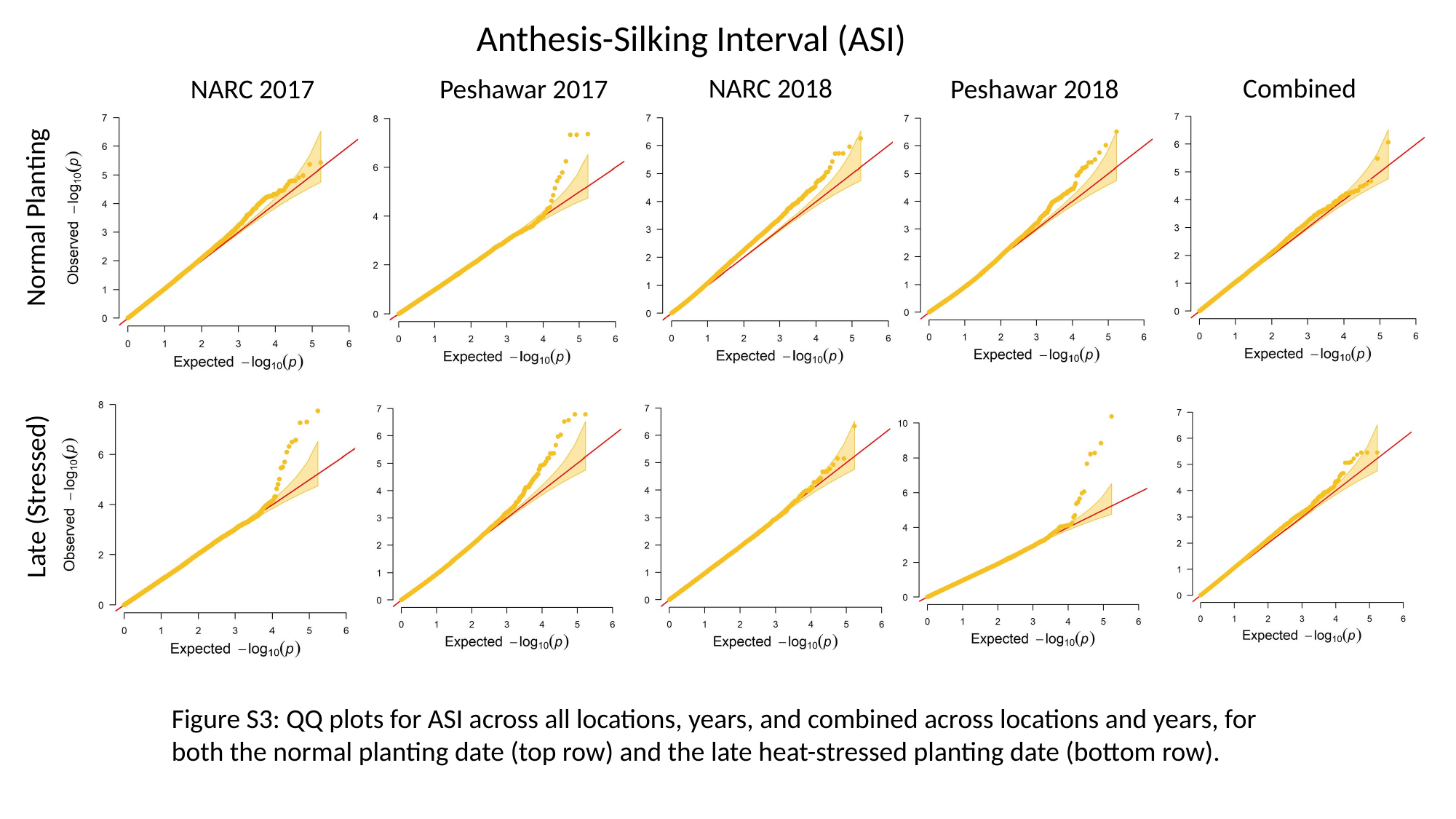

Anthesis-Silking Interval (ASI)
NARC 2018
Combined
NARC 2017
Peshawar 2017
Peshawar 2018
Normal Planting
Late (Stressed)
Figure S3: QQ plots for ASI across all locations, years, and combined across locations and years, for both the normal planting date (top row) and the late heat-stressed planting date (bottom row).

## Slide 4
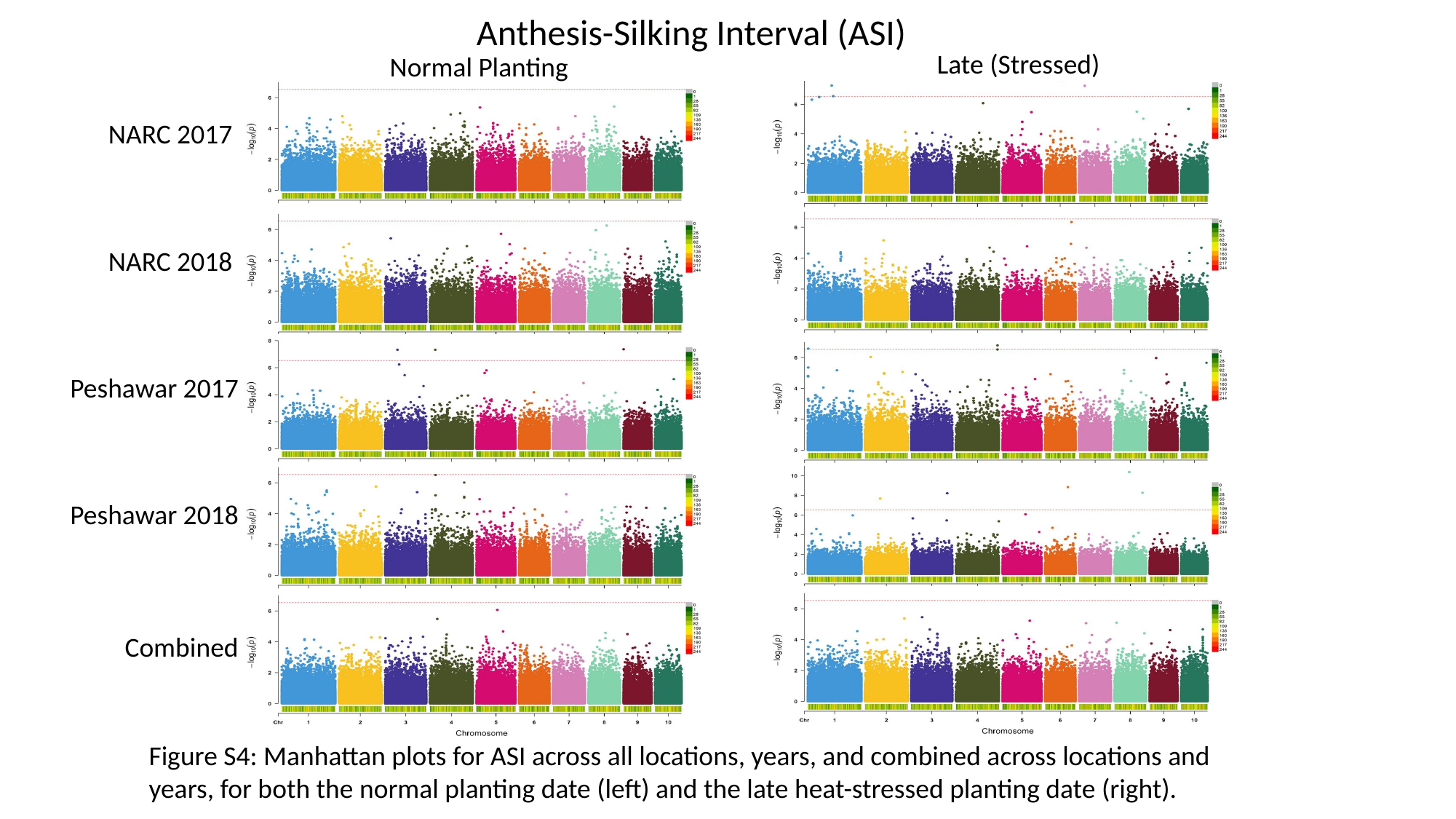

Anthesis-Silking Interval (ASI)
Late (Stressed)
Normal Planting
NARC 2017
NARC 2018
Peshawar 2017
Peshawar 2018
Combined
Figure S4: Manhattan plots for ASI across all locations, years, and combined across locations and years, for both the normal planting date (left) and the late heat-stressed planting date (right).
